# The Humidity in a Low-Flow Dräger Fabius Anesthesia Workstation with or without Thermal Insulation or a Heat and Moisture Exchanger: A Prospective Randomized Clinical Trial

**DOI:** 10.1371/journal.pone.0170723

**Published:** 2017-01-27

**Authors:** Sergius A. R. de Oliveira, Lorena M. C. Lucio, Norma S. P. Modolo, Yoko Hayashi, Mariana G. Braz, Lídia R. de Carvalho, Leandro G. Braz, José Reinaldo C. Braz

**Affiliations:** 1 Department of Anesthesiology, Botucatu Medical School, UNESP–Univ Estadual Paulista, Botucatu, Sao Paulo State, Brazil; 2 Department of Biostatistics, Institute of Biosciences, UNESP–Univ Estadual Paulista, Botucatu, Sao Paulo State, Brazil; University of Colorado, UNITED STATES

## Abstract

**Background:**

During anesthesia, as compared with intensive care, the time of the tracheal intubation is much shorter. An inhaled gas minimum humidity of 20 mgH_2_O.L^-1^ is recommended to reduce the deleterious effects of dry gas on the airways during anesthesia with tracheal intubation. The Fabius GS Premium® anesthesia workstation (Dräger Medical, Lübeck, Germany) has a built-in hotplate to heat gases in the breathing circuit. A heat and moisture exchanger (HME) is used to further heat and humidify the inhaled gas. The humidity of the gases in the breathing circuit is influenced by the ambient temperature. We compared the humidity of the inhaled gases from a low-flow Fabius anesthesia workstation with or without thermal insulation (TI) of the breathing circuit and with or without an HME.

**Methods:**

We conducted a prospective randomized trial in 41 adult female patients who underwent elective abdominal surgery. The patients were allocated into four groups according to the devices used to ventilate their lungs using a Dräger Fabius anesthesia workstation with a low gas flow (1 L.min^-1^): control, with TI, with an HME or with TI and an HME (TIHME). The mean temperature and humidity of the inhaled gases were measured during 2-h after connecting the patients to the breathing circuit.

**Results:**

The mean inhaled gas temperature and absolute humidity were higher in the HME (29.2±1.3°C; 28.1±2.3 mgH_2_O·L^-1^) and TIHME (30.1±1.2°C; 29.4±2.0 mgH_2_O·L^-1^) groups compared with the control (27.5±1.0°C; 25.0±1.8 mgH_2_O·L^-1^) and TI (27.2±1.1°C; 24.9±1.8 mgH_2_O·L^-1^) groups (*P* = 0.003 and *P*<0.001, respectively).

**Conclusions:**

The low-flow Fabius GS Premium breathing circuit provides the minimum humidity level of inhaled gases to avoid damage to the tracheobronchial epithelia during anesthesia. TI of the breathing circuit does not increase the humidity of the inhaled gases, whereas inserting an HME increases the moisture of the inhaled gases closer to physiological values.

## Introduction

In physiological conditions, inhaled air is warmed and humidified while passing through the nose and upper airways, reaching the subglottic space with a temperature of 31.2°C to 33.6°C, a relative humidity (RH) of 95%-100% and an absolute humidity (AH) of 33 mg H_2_O.L^-1^[1–3]. However, these functions are bypassed when a patient’s trachea is intubated or a supraglottic airway device is placed in situ [4]. In this situation, ventilation with dry and cold compressed gases leads to considerable water and heat loss from the respiratory tract [4–9]. Upper airways dehydration may lead to reduced mucociliary transport, cilia and mucous gland destruction and thickened secretions [3,4,7–10]. Additionally, alterations in pulmonary function associated with inadequate humidity can occur, including reduced residual capacity, decreased pulmonary compliance, and increased pulmonary shunt, hypoxemia and atelectasias [3,9]. Hence, during tracheal intubation with mechanical ventilation, the gas delivered to the patient must be artificially conditioned to replace these lost functions [11–16].

During anesthesia, as compared with intensive care, the time of the tracheal intubation is much shorter. In a model of moisture deficit and duration of exposure on dysfunction of the respiratory tract, it was demonstrated that lower levels of humidity could be tolerated for short period of time without causing dysfunction [3]. According the literature data, 20 mg H_2_O.L^-1^ is an appropriate minimum target for moisture output for use in general anesthesia to avoid respiratory tract dehydration [9,10–12].

The Dräger Fabius GS Premium® anesthesia workstation (Dräger Medical, Lübeck, Germany) has a built-in hotplate to heat gases in the inspiratory and expiratory branches of the breathing circuit. The hotplate could likely warm the gases inhaled from this anesthesia machine, but the warming and humidifying properties of this workstation have not yet been investigated.

During anesthesia, the circle breathing system with carbon dioxide (CO_2_) absorber has humidifying properties due to the partial rebreathing of humidified exhaled gases and generation of water and heat associated with the reaction of CO_2_ with the soda lime. The use of a low fresh gas flow (FGF– 1 L.min^-1^) in the circuit increases the humidity of the inhaled gas because more humidified exhaled gases are rebreathed [12,13].

A heat and moisture exchanger (HME) is a device used to further heat and humidify the inhaled gases during anesthesia [11,14–17]. HMEs operate passively by storing heat and moisture from the patient`s exhaled gases and releasing heat and moisture with the inhaled gases [11].

The operating room (OR) temperature directly influences the heat and moisture of the inhaled gases at different anesthesia workstations [14,18]. The thermal insulation (TI) of the breathing circuit increased the temperature and humidity of the inhaled gases in an experimental model because the corrugated tubes of the breathing circuit comprise materials with low TI [18].

This study aimed to evaluate the mean temperature and humidity of the inhaled gases from a low-flow breathing system of the Dräger Fabius GS Premium® anesthesia workstation and to compare the effects of adding TI or an HME to the breathing circuit on the mean temperature and humidity of the inhaled gases from this anesthesia workstation.

## Patients and Methods

**Fig 1:**
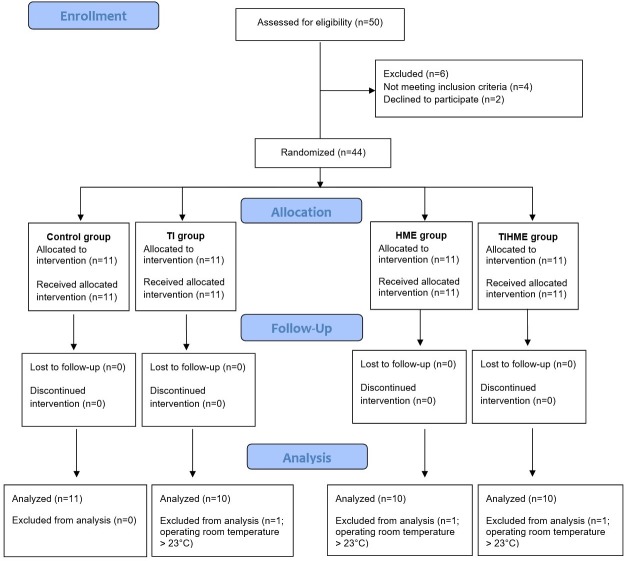
Flow diagram of the study. TI = thermal insulation; HME = heat and moisture exchanger; TIHME = thermal insulation + heat and moisture exchanger.

This study was performed at Sao Paulo State University Hospital (UNESP, Botucatu, Brazil) and was approved by the Institutional Review Board (Human Research Ethics Committee of the Botucatu Medical School-UNESP, #4080/2011) on December 5, 2011. The protocol study could only be sent to the Brazilian Clinical Trials Registry (ReBEC) on April 11, 2013, since the ReBEC site was facing a series of operational problems, postponing the trial registers. Thus, our study was only registered on June 11, 2015 (RBR-26ssvc). The date for patient recruitment was from January 30, 2012 to November 21, 2013. Thus, we confirm that the related trial for the interventions is registered. Written informed consent was obtained from all of the patients studied. We studied 44 adult (18 to 64 years old), afebrile (T < 37°C) women with a body mass index from 20 to 30 kg.m^-2^, and ASA physical status I or II scheduled for elective open abdominal gynecologic surgery (hysterectomy or oophorosalpingectomy) with an anticipated anesthesia duration of 2 hours or longer (Fig 1). Patients with any pulmonary or cardiac disease or with a body mass index > 30 kg.m^-2^ were excluded from the study.

### Study design

In the OR, the patients were randomly allocated by sequentially numbered sealed envelope assignment into four groups (11 women per group), according to the use of a low-flow breathing circuit with TI or an HME, as follows: control group, without TI or HME; TI group, with TI and without HME; HME group, without TI and with HME; and TIHME group, with TI and HME. An anesthesiologist who was not involved in the perioperative management of the patients printed 44 group identification tags (11 for each group) and placed these tags into 44 opaque envelopes (one identification tag per envelope). The same anesthesiologist then sealed, mixed and sequentially numbered the envelopes in ascending order. Just before each anesthesia induction, one envelope was opened following the sequential order to identify the study group.

A Dräger Fabius GS Premium anesthesia workstation was used in all cases. Before each anesthetic procedure, the branches of the anesthesia breathing system was replaced by clean and dry silicone corrugated tubes of the same length (1.5 m; Dräger Medical, Lübeck, Germany). The CO_2_ canister (1.5 L) of the anesthesia machine was filled with fresh soda lime (Drägersorb 800 Plus, Dräger, Lübeck, Germany) before each case. The breathing circuit, including the CO_2_ absorber, had an internal volume of 4.5 L. In the TI and TIHME groups, the branches of the breathing circuit were covered by three layers of aluminum sheets. In the HME and TIHME groups, a hygroscopic HME (Venticaire model 038-41-355; Flexicare Medical Limited, Mountain Ash, UK) was placed between the Y-piece of the breathing circuit and the tracheal tube. According to the manufacturer, this HME has 29 g weight, 69 ml of dead space and 0.9 cm H_2_O of resistance at 30 L.min^-1^.

### Anesthesia protocol

Upon arrival to the OR, and after 8 hours of fasting, the patients received standard clinical monitoring with an electrocardiogram (D_II_ and V_5_ leads), peripheral oxygen saturation (SpO_2_), noninvasive arterial blood pressure measurements, a neuromuscular blockade monitor (TOF-watch SX, GPV Elbau Electronics A/S, Aars, Denmark) and a cerebral state index (CSI®) (Danmeter, Biometer International, Odense, Denmark).

An intravenous (IV) line was inserted with a 20 or 18-G catheter and the patients received 3 mg midazolam via IV. Fluid deficits were replaced with lactated Ringer’s solution at 10 mL.kg^-1^.h^-1^. In all of the patients, the fluids were maintained at the OR temperature. All of the patients received active skin-surface warming with a specific blanket on the lower limbs using a warming device (Bair Hugger®, model 750, Arizant Healthcare, Minneapolis, MN) set to delivery forced-air at 43°C following anesthesia induction and lasting until the end of the surgery.

Total intravenous anesthesia with propofol and remefentanil was utilized in all patients. An IV bolus of cisatracurium besylate (0.15 mg.kg^-1^) was given to facilitate orotracheal intubation.

After tracheal intubation, a FGF of 2.0 L.min^-1^ (1.0 L.min^-1^ of O_2_ in 1.0 L.min^-1^ of air) was supplied to the circle breathing during the first 5 minutes and then adjusted to 1.0 L.min^-1^ (0.5 L.min^-1^ of O_2_ in 0.5 L.min^-1^ of air). The lungs were mechanically ventilated using the volume-controlled mode of the Fabius anesthesia workstation with a tidal volume of 8 mL.kg^-1^. The respiratory rate was adjusted to maintain an end-tidal carbon dioxide (PetCO_2_) pressure of approximately 35 mm Hg. The inspiratory and expiratory oxygen concentrations, PetCO_2_ and ventilation variables were monitored with the Fabius built-in monitor.

### Measurement of temperature and humidity of the gases

**Fig 2:**
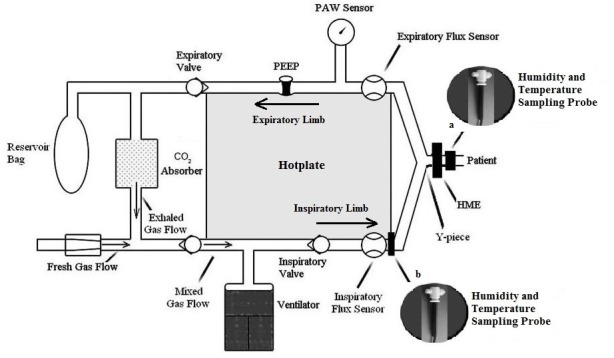
A diagram of the Fabius GS Premium breathing circuit. The humidity and temperature sensor was placed between the Y-piece and the tracheal tube or between the heat and moisture exchanger (HME) and the tracheal tube (position a) when used to measure inhaled gas temperature and humidity; and it was placed in the inspiratory limb outlet close to the anesthesia workstation (position b) when used to measure the temperature and humidity of the inspired gas limb outlet. PEEP = positive end-expiratory pressure; PAW = airways pressure.

The primary outcomes temperature and humidity of the gases were intermittently measured using a rapidly responding electronic digital thermo-hygrometer (Vaisala Humicap® Hand-Held Humidity and Temperature Meter HM 70, Helsinki, Finland) that was connected by a T-piece between the Y-piece of the breathing circuit and the tracheal tube in the control and TI groups, and between the HME and the tracheal tube in the HME and TIHME groups (Fig 2, position a); and it was connected to the inspiratory limb outlet close to the anesthesia workstation in all of the groups (Fig 2, position b). The thermo-hygrometer operates on a capacitive principle and has a reported accuracy of ± 2.0% for RH and ± 0.2°C for temperature. The temperature and humidity of the gases fluctuated with the respiratory cycle phases and were lower in the inspiratory phase. We recorded the minimal temperature and RH values averaged over 4 respiratory cycles after 10, 30, 60, 90, and 120 minutes of connection between the patient and the respiratory circuit after tracheal intubation. The AH values were calculated using the thermo-hygrometer software with the formula AH = (3.939 + 0.5019T + 0.00004615T^2^ + 0.0004188T^3^) × RH/100, in which AH is the absolute humidity (mg H_2_O.L^-1^), T is the temperature (°C), and RH is the relative humidity (%).

The secondary outcome intraoperative distal esophageal (core) temperature was measured after tracheal intubation using a thermocouple sensor (Mon-a-therm 90,044®, Mallinckrodt Medical, Veracruz, Mexico). The thermocouple sensors for the esophagus and OR temperatures were attached to a 2-channel electronic thermometer (4070, Mallinckrodt Medical, St. Louis, MO).

Postoperative analgesia comprising 0.1 mg.kg^-1^ morphine, 100 mg tramadol and 100 mg cetoprofen IV was provided 15 minutes before the end of the surgery. The residual neuromuscular blockade was reversed with 30 μg.kg^-1^ of neostigmine and 10 μg.kg^-1^ of atropine IV, if necessary. Tracheal extubation was performed after full reversal of the neuromuscular blockade. All of the patients were immediately transferred to the Postanesthesia Care Unit.

### Statistical analysis

The sample size of the groups was calculated based on data found in the literature regarding the humidification of gases during anesthesia [7,19] and assuming an AH minimum detectable difference of 5.0 mg H_2_O.L^-1^ among the groups, with an expected standard deviation (SD) of residuals of ± 3.0 mg H_2_O.L^-1^, as significant. For 80% power and considering the risk of a type I error as α of 0.05 or less and of committing type II error as β of 0.20 or less, 9 patients in each group would be required. Since missing data of patients can occur during the study, 11 patients in each group were enrolled.

The normal distribution of the data was confirmed using Lilliefors tests. The pooled data from each of the four groups followed a normal distribution (all Lilliefors *P* > 0.10). The anthropometric variables were compared among groups by analysis of variance (ANOVA). The AH, RH, and temperature values were compared among the groups at different time points by repeated measured ANOVA (Profile Analysis). In this analysis, the following hypotheses were tested: there was no interaction between groups and time points, and there was no difference between mean groups over time. This analysis was followed by Tukey’s test for pairwise comparisons. Pearson’s coefficient was used for the correlation analysis between the OR and inhaled gas temperatures in all of the groups.

The data were expressed as the means ± SD and 95% confidence intervals (CIs). The statistical analyses were performed using the Statistical Package for the Social Sciences (Windows Software, version 17.1; SPSS Inc., Chicago, IL). For all analyses, *P* < 0.05 was considered statistically significant.

## Results

Forty-four patients were enrolled in the study, but critical data were missing (OR temperature > 23°C) for three patients: one patient in the TI group, one in the HME group and one in the TIHME group. The remaining 41 patients were included in the statistical analyses, giving 11 patients in the control group and 10 in each of the remaining groups (Fig 1).

No significant differences were found among the groups with regard to patient characteristics or anesthesia length (Table 1), hemodynamic variables (data not shown), and mean OR or core (esophageal) temperatures (Table 2).

**Table 1:** Patient Characteristics and Anesthesia Length Data in the Studied Groups.

Groups	Age (years)	Weight (kg)	Height (cm)	Anesthesia Length (cm)
Control	44 ± 11	70.0 ± 12.1	160.5 ± 5.9	186 ± 50
TI	47 ± 7	76.8 ± 7.5	159.7 ± 4.9	202 ± 48
HME	46 ± 7	77.1 ± 9.0	160.5 ± 8.2	158 ± 51
TIHME	50 ± 6	74.2 ± 13.0	160.3 ± 5.3	163 ± 21
*P* value	0.43	0.40	0.99	0.12

Data expressed as means ± standard deviations.

TI = thermal insulation; HME = heat and moisture exchanger; TIHME = thermal insulation + heat and moisture exchanger.

**Table 2:** Operating Room and Core Temperature Data in the Studied Groups.

Temperature (°C)	Groups	Mean over time	95% CI	*P* value	
				Difference among groups	Time x group interaction
Operating room	Control	**21.3 ± 0.9**	21.0–21.5		
	TI	**21.3 ± 1.6**	20.8–21.7		
	HME	**21.7 ± 1.0**	21.4–22.0	0.32	0.90
	TIHME	**22.0 ± 1.0**	21.7–22.3		
Core	Control	**35.8 ± 0.3**	35.8–35.9		
	TI	**36.0 ± 0.5**	35.9–36.2		
	HME	**36.1 ± 0.5**	35.9–36.2	0.29	0.039
	TIHME	**35.8 ± 0.3**	35.7–35.9		

Data expressed as means ± standard deviations.

Mean over time: the mean of each measurement within each subject from 10 to 120 min after connection of the patients to the breathing circuit; the standard deviation (SD) listed is the SD of the means among the subjects.

CI = confidence interval; TI = thermal insulation; HME = heat and moisture exchanger; TIHME = thermal insulation + heat and moisture exchanger.

The mean gas temperature in the inspiratory limb outlet of the workstation was not different among the groups (*P* = 0.87; Table 3). The mean temperature values of the inhaled gases were significantly higher in the HME and TIHME groups compared with the control and TI groups (*P* = 0.003; Table 3). The differences between the mean inhaled and workstation inspiratory limb gas temperatures were significantly higher in the HME and TIHME groups compared with the control and TI groups (*P* < 0.001; Table 3). The mean RH and AH of the gases in the inspiratory limb outlet close to anesthesia workstation did not differ among the groups (*P* = 0.06 and *P* = 0.07, respectively; Table 4). The mean RH and AH values of the inhaled gases were significantly higher in the HME and TIHME groups compared with the control and TI groups (*P* < 0.001; Table 5).

**Table 3:** Workstation Outlet Inspiratory Limb and Inhaled Gas Temperature Data in the Studied Groups.

Temperature (°C)	Groups	Mean over time	95% CI	*P* value	
				Difference among groups	Time × group interaction
Workstation outlet inspiratory limb gas	Control	**28.3 ± 0.9**	28.0–28.5		
	TI	**28.3 ± 1.3**	27.9–28.7		
	HME	**28.2 ± 1.6**	27.7–28.6	0.87	0.039
	TIHME	**28.0 ±1.1**	27.7–28.3		
Inhaled gas	Control	**27.5 ± 1.0 B**	27.2–27.8		
	TI	**27.2 ± 1.1 B**	26.8–27.5		
	HME	**29.2 ± 1.3 A**	28.8–29.6	0.003	0.95
	TIHME	**30.1 ± 1.2 A**	29.8–30.5		
Workstation outlet inspiratory limb and inhaled gas temperature difference	Control	**- 0.8 ± 0.8 B**	- 1.0; - 0.5		
	TI	**- 1.2 ± 1.6 B**	- 1.6; - 0.7		
	HME	**1.0 ± 2.0 A**	0.5; 1.6	< 0.001	0.28
	TIHME	**2.1 ± 1.5 A**	1.7; 2.5		

Data expressed as means ± standard deviations. Mean over time: the mean of each subject within each group from 10 to 120 min after connection of the patients to the breathing circuit; the standard deviation (SD) listed is the SD of the means among the subjects.

CI = confidence interval; TI = thermal insulation; HME = heat and moisture exchanger; TIHME = thermal insulation + heat and moisture exchanger.

The means followed by different letters are significantly different.

**Table 4:** Relative and Absolute Humidity Data of the Gases in the Workstation Outlet Inspiratory Limb in the Studied Groups.

	Time (min)							*P* value	
Workstation outlet inspiratory limb gas/ Groups	10	30	60	90	120	Mean over time	95% CI	Difference among groups	Time × group interaction
**RH (%)**									
Control	61.2 ± 11.6	62.3 ± 9.9	66.7 ± 9.6	71.3 ± 10.1	73.5 ± 7.3	**67.0 ± 10.6**	64.0–70.0		
TI	63.7 ± 7.7	63.0 ± 6.0	71.3 ± 7.5	80.6 ± 7.9	80.7 ± 9.6	**71.9 ± 10.8**	68.6–75.1	0.06	0.32
HME	62.3 ± 12.1	67.1 ± 10.9	74.7 ± 11.7	80.7 ± 11.4	83.6 ± 8.4	**73.7 ± 13.3**	69.7–77.6		
TIHME	68.9 ± 9.0	70.5 ± 8.5	75.9 ± 8.6	83.1 ± 10.7	85.6 ± 11.4	**76.8 ± 11.5**	73.4–80.2		
**AH (mg H**_**2**_**O·L**^**-1**^**)**									
Control	17.2 ± 3.9	17.2 ± 2.8	18.4 ± 2.5	19.5 ± 2.8	20.2 ± 2.1	**18.5 ± 3.0**	17.6–19.4		
TI	18.0 ± 1.2	17.8 ± 1.4	19.6 ± 2.0	21.4 ± 1.5	22.2 ± 1.8	**19.8 ± 2.4**	19.1–20.5	0.07	0.54
HME	18.4 ± 3.5	18.4 ± 2.1	19.8 ± 2.0	21.7 ± 2.9	22.1 ± 2.0	**20.1 ± 2.9**	19.2–21.0		
TIHME	19.1 ± 2.5	18.8 ± 2.6	20.5 ± 2.8	22.4 ± 3.1	23.9 ± 3.0	**20.9 ± 3.3**	20.0–21.9		

Data expressed as means ± standard deviations. Mean over time: the mean of each measurement within each subject from 10 to 120 min after connection of the patients to the breathing circuit; the standard deviation (SD) listed is the SD of the means among the subjects.

CI = confidence interval; RH = relative humidity; AH = Absolute humidity; TI = thermal insulation; HME = heat and moisture exchanger; TIHME = thermal insulation + heat and moisture exchanger.

**Table 5:** Relative and Absolute Humidity of the Inhaled Gases in the Studied Groups.

	Time (min)							*P* value	
Inhaled Gases/ Groups	10	30	60	90	120	Mean over time	95% CI	Difference among groups	Time × group interaction
**RH (%)**									
Control	93.8 ± 4.6	91.0 ± 3.2	95.9 ± 2.7	93.7 ± 4.0	92.9 ± 3.3	**93.5 ± 3.8 B**	92.4–94.5		
TI	94.8 ± 5.6	95.2 ± 3.2	95.9 ± 3.3	94.5 ± 3.5	96.5 ± 2.9	**95.4 ± 3.7 B**	94.3–96.5	< 0.001	0.10
HME	96.0 ± 3.8	95.4 ± 2.8	96.2 ± 1.9	97.5 ± 1.4	96.5 ± 1.4	**96.3 ± 2.4 A**	95.6–97.0		
TIHME	95.6 ± 3.1	95.7 ± 2.2	96.9 ± 2.7	95.8 ± 2.0	97.1 ± 1.7	**96.2 ± 2.4 A**	95.5–96.9		
**AH (mg H**_**2**_**O·L**^**-1**^**)**									
Control	24.7 ± 2.4	24.6 ± 1.9	25.2 ± 1.3	25.1 ± 1.7	25.2 ± 1.8	**25.0 ± 1.8 B**	24.4–25.5		
TI	24.5 ± 1.8	24.7 ± 1.7	24.8 ± 2.3	24.7 ± 1.9	25.6 ± 1.3	**24.9 ± 1.8 B**	24.3–25.4	< 0.001	0.31
HME	28.6 ± 3.1	28.0 ± 2.2	28.0 ± 2.3	28.6 ± 2.2	27.5 ± 2.1	**28.1 ± 2.3 A**	27.4–28.8		
TIHME	29.0 ± 1.4	28.9 ± 2.0	29.5 ± 1.9	29.5 ± 2.3	30.1 ± 2.3	**29.4 ± 2.0 A**	28.8–30.0		

Data expressed as means ± standard deviations. Mean over time: the mean of each subject within each group from 10 to 120 min after connection of the patients to the breathing circuit; the standard deviation (SD) listed is the SD of the means among the subjects.

CI = confidence interval; RH = relative humidity; AH = absolute humidity; TI = thermal insulation; HME = heat and moisture exchanger; TIHME = thermal insulation + heat and moisture exchanger.

The means followed by different letters are significantly different.

A significant and positive correlation was found between the OR and inhaled gas temperatures in the control group (*r* = 0.53; *P* < 0.001) but not in the TI (*r* = 0.12; *P* = 0.40), HME (*r* = 0.15; *P* = 0.30) or TIHME (*r* = 0.12; *P* = 0.40) groups.

None of the patients had any surgical or clinical problems, and all of the patients were discharged from the hospital, according to the guidelines and protocols established for their particular surgical procedures.

## Discussion

There were three main findings in this study: (1) the minimum recommended inhaled gas humidity of 20 mg H_2_O.L^-1^ during anesthesia was achieved in all groups since the first measurement; (2) the use of TI in the breathing circuit did not significantly increase the temperature or moisture of the inhaled gases; and (3) the use of an HME increased the temperature and humidity of the inhaled gases closer to physiological values.

During general anesthesia, the two sources of heat and moisture in a circle breathing circuit are the rebreathing of exhaled gas, and the water vapor and heat released from the CO_2_ absorbent in an exothermic reaction [20]. The conservation of heat and moisture in the breathing circuit depends on various factors, including the FGF, the breathing system configuration, and the OR temperature.

Increased rebreathing of exhaled gases is expected with a lower FGF; consequently, higher temperature and moisture of the inhaled gases are also expected. Some studies have demonstrated that a low FGF (1 L.min^-1^) increased the temperature and moisture of the inhaled gas compared with a high FGF (≥ 3.0 L.min^-1^) [12,13,19,21]. However, no significant differences in inhaled gas temperature and humidity were found by using the minimum FGF (0.5 L.min^-1^) compared with a low FGF during mechanical ventilation in adult patients undergoing general anesthesia [22,23].

In the Fabius GS Premium anesthesia workstation breathing system, the exhaled gases move through the hotplate and cross the soda lime once before mixing with the cold and dry FGF. The mixed gases are then pulled by the plunger to fill the ventilator. After opening the inspiratory valve, the ventilator plunger sends the gaseous mixture to the inspiratory limb of the respiratory circuit where it is warmed a second time by the built-in hotplate (Fig 2). The Primus anesthesia workstation (Dräger, Lübeck, Germany) also has a built-in hotplate; however, unlike the Fabius GS Premium, only the expiratory limb is warmed [14,21]. A study using this machine with a low-flow breathing system showed a lower temperature (25.3 ± 1.4°C) and AH (20.5 ± 3.6 mg H_2_O.L^-1^) of the inspired gas after 120 minutes of connection of the patient to the breathing circuit, compared with our data [14]. Other anesthetic workstations, such as the Cicero and Cato (Dräger, Lübeck, Germany), also have a built-in hotplate to heat the exhaled gases. However, unlike the Fabius, the exhaled gases pass through a heated, wet canister and, after mixing with a dry, cold FGF, pass through the CO_2_ absorber twice per breath [22,23]. In the studies using these anesthesia workstations with a low FGF, the inhaled gas AH values were higher (≥ 27.5 mg H_2_O.L^-1^) compared with our data [22,23]. However, in these studies, the OR temperatures were higher (approximately 3–4°C) compared with our data, which may have influenced the inhaled gas AH values.

Our data regarding the temperature and moisture of the inhaled gases with a low FGF were higher compared with the other conventional non-heated, low-flow anesthesia machines, such as the Aestiva/5 (Datex-Ohmeda, Helsinki, Finland) [23], Ohmeda Excel 210 (Helsinki, Finland) [24] and Nikkei (K. Takaoka, São Paulo, Brazil) [7].

In short-duration surgeries, an anesthetic breathing circuit that properly humidifies the inhaled gases should be effective early in the procedures. The heated anesthetic breathing system from the Dräger Fabius achieved the minimum recommended inhaled gas humidity during anesthesia since the first measurement (10 minutes).

In our study, a significant and positive correlation was found between the OR and inhaled gas temperatures only in the control group. Thus, the use of corrugated tubes with TI could increase the temperature of the inhaled gases from the Fabius machine. However, our data showed similar mean temperatures of the inhaled gases in the control and TI groups; additionally, the differences between the mean temperatures of the gases of the workstation inspiratory limb and the inhaled gases were small (approximately 1°C) and did not differ between the control and TI groups and between the HME and TIHME groups. The speed and amount of heat loss are directly related to the heat transfer coefficient of the material and the length of the inspiratory limb of the breathing circuit [25]. In some studies performed with a low FGF and silicone corrugated tubes in the breathing circuit, the inhaled gas temperature was lower (approximately 4°C) than the workstation inspiratory limb gas temperature [14,22]. We also utilized low FGF and silicone corrugated tubes. Thus, the small temperature decrease of the gases in the inspiratory circuit of the Dräger Fabius demonstrated the effectiveness of its hotplate to heat gases in the inspiratory branch of the breathing circuit, which was likely the main factor responsible for not obtaining higher temperature values of the inhaled gases in the TI groups compared with the non-TI groups.

During anesthesia, as compared with intensive care, the time of the tracheal intubation is much shorter. Therefore, as emphasized in a review on the HME use in anesthesia and intensive care [11], the additional use of an HME during the anesthesia is probably not required when the minimum humidity level of the inhaled gases is provided by the anesthesia workstation. In addition, in a porcine model, it was verified that a minimum level of 20 mg H_2_O.L^-1^ of AH was sufficient to prevent damage to tracheobronchial epithelia during 10 h of mechanical ventilation [12].

Williams et al.’s model of humidity deficit and duration of exposure on the respiratory tract according to the individual's state of health showed that the critically ill patient is less tolerant to water mass and thermal challenges to their airway mucosa [3]. Therefore, the use of an HME in the anesthetic breathing circuit in a low-flow Fabius GS Premium machine is indicated to patients with respiratory tract dysfunction or who need a postoperative long-term mechanical ventilation.

No minimum requirement for humidification performance in the draft standard for HMEs has been established [26]. Our data of the mean AH values of the inhaled gases in the HME (28.1 ± 2.3 mg H_2_O.L^-1^) and TIHME (29.4 ± 2.0 mg H_2_O.L^-1^) groups were closer to those required by the American Association of Respiratory Care (30 mg H_2_O.L^-1^) for the safe and effective performance of HMEs for long-term mechanical ventilation in patients whose upper airways have been bypassed by an endotracheal tube [27]. In agreement with our data, many studies obtained similar AH values of the inhaled gases with an HME in the breathing circuit using low-flow anesthesia workstations [14,15,21,23,24].

Many patients in all of the groups had mild intraoperative hypothermia. The use of active skin-surface warming may have minimized heat loss but did not avoid core hypothermia resulting from an internal core-to-peripheral redistribution of heat after induction of general anesthesia [28]. Although the inhaled gas temperature was increased by the Fabius hotplate and the addition of an HME, respiration did not influence the core temperature; less than 10% of metabolic heat production is lost through the respiration [29]. In one study, the use of an HME alone did not prevent intraoperative hypothermia in adults [19].

A limitation of this study is that it was not blinded, which could result some bias. The gas humidity and temperature measurements were made intermittently at predefined times. In the HME groups, the RH and temperature sensor of the thermo-hygrometer was connected by a T-piece between the tracheal tube and the HME; because of the proximity, concealing the presence of the HME was not possible during the measurements. Only women were included in our study. This could be an important concern. However, at this time, we have no knowledge of any study that suggests gender differences with regard to heating and humidification of upper airways. Thus, the results obtained with women could be extrapolated to male population.

More studies are necessary to verify if the breathing circuit with built-in hotplate from Dräger Fabius anesthesia workstation reaches the minimum humidity level of the inhaled gases under the conditions of a FGF ≥ 2 L.min^-1^ or a low FGF associate with low minute volume (< 5 L.min^-1^). The effects of adding TI to the breathing circuit from other low-flow anesthesia workstations with built-in hotplate, such as Dräger Primus, on the temperature and humidity of the inhaled gases also need to be investigated.

## Conclusions

In adult patients, the use of a low FGF in the Fabius GS Premium breathing circuit provides the minimum humidity level of the inhaled gases to avoid damage to the tracheobronchial epithelia during anesthesia. TI of this breathing circuit does not increase the temperature and humidity of the inhaled gases, whereas the insertion of an HME in the anesthetic breathing circuit increases both the temperature and the humidity of the inhaled gases closer to required values to surgical patients with respiratory tract diseases or who need postoperative controlled ventilation.

## Supporting Information

S1 Fig CONSORT Checklist. (DOC)

S1 Protocol English Study Protocol. (DOC)

S2 Protocol Portuguese Study Protocol. (DOC)
