# Supplementary material for: The Humidity in a Low-Flow Dräger Fabius Anesthesia Workstation with or without Thermal Insulation or a Heat and Moisture Exchanger: A Prospective Randomized Clinical Trial
Source: PLoS One. 2017 Jan 27;12(1):e0170723. doi: 10.1371/journal.pone.0170723 (PMC5271343; doi:10.1371/journal.pone.0170723)
Supplement: S1 Protocol — (DOC) [file pone.0170723.s002.doc]

**PROTOCOL STUDY SENT TO HUMAN RESEARCH ETHICS COMMITTEE OF THE BOTUCATU MEDICAL SCHOOL-UNESP**

**The Humidity in a Low-Flow Dräger Fabius Anesthesia Workstation With or Without Thermal Insulation or a Heat and Moisture Exchanger**

**Sergius Arias Rodrigues de Oliveira**

Post-Graduation Program in Anesthesiology, Botucatu Medical School, UNESP- Univ. Estadual Paulista

**José Reinaldo Cerqueira Braz**

Full Professor, Department of Anesthesiology, Botucatu Medical School, UNESP- Univ. Estadual Paulista

**October, 2011**

**1-INTRODUCTION**

 In physiological conditions, the upper airways heat and humidify the inhaled air. During the inspiration, the air reaches the alveoli at body temperature (37°C) with 100% relative humidity (RU) and 44 mg H_2_O.L^-1^ absolute humidity (AH)^1^. The fresh gas flow (FGF) used during anesthesia is cold and dry. During tracheal intubation, the functions of the nose and the other upper airways are bypassed. If the humidification and heating of inhaled gases are not adequate, functional impairment occurs in the airway mucosa, by changing the cilia movement and increasing the mucus viscosity, which can lead to cilia and mucous destruction, increased airway resistance, changes in ventilation-perfusion ratio, microatelectasias, decreased lung compliance and functional residual capacity due to the changes of pulmonary surfactant, besides inflammatory reactions, ulcerations in the mucous membrane and dehydration, predisposing the formation of plugs that block the airways and the tracheal tube^2-6^. Thus, during artificial ventilation under tracheal intubation, heating and humidifying of the inspired gases are essential to ensure the integrity of the airways and the preservation of mucociliary function and gas exchange^4,7^.

There is disagreement regarding the ideal values of heating and humidification of inhaled gases, but it is necessary to establish limits of heating and humidification of the inhaled gases to prevent mucosal respiratory lesions^8^. An experimental study, performed in dogs who underwent tracheal intubation and mechanical ventilation for three hours, showed the effects of warming and humidifying inspired gases, according to the histological changes of the mucosal epithelium of the tracheobronchial tree to optical microscopy. The authors concluded that the temperature and AH of inspired gases must not exceed 36°C and 36 mg H_2_O.L^-1^, respectively, and should not have temperature below 27°C or AH below 23 mg H_2_O. L^-1 9^.

The use of low FGF during inhalation anesthesia has increased in recent years due to its important advantages, such as lower inhaled anesthetics consumption, more effective heating and humidification of the inspired gas and significant reduction of environmental pollution^10-12^. However, the use of low FGF has disadvantages that include the need for greater attention and anesthesiologist's care due to the difficult rapid change of the inspired concentration of inhaled anesthetic, most likely to hypercarbia production by the quickest exhaust system carbon dioxide absorption and possible accumulation of undesirable gases such as carbon monoxide, acetone, methane, and toxic metabolites of anesthetic agents^12^ requiring periodic "washing" of the breathing system at high FGF for a few minutes.

The reduction in the FGF leads to better utilization of heat and moisture from exhaled air and that is generated in the canister reservoir, through the CO_2_ neutralization reaction by soda lime, which is exothermic and leads to water training^13-15^. Several authors used low FGF with variable efficacy in relation to the heat and moisture inhaled gases levels^10,14-17^, especially when combined with the addition of a heat and moisture exchanger (HME) in the breathing circuit^10,14^. The different results in the studies maybe explained by different configurations of the breathing systems of the anesthesia machines, the different methods used for measuring moisture in the inspired gases, the different performances of the HMEs^18, 19^ and the FGF rate used^10^.

The operating room (OR) temperature directly influences the heat and moisture of the inhaled gases at different anesthesia workstations due to the low thermal insulation (TI) of silicone tubing used in the anesthetic breathing circuit^14,15^.

The Fabius GS Premium anesthesia workstation (Dräger Medical, Lübeck, Germany) has a built-in hotplate to heat the gases in the breathing circuit. In this anesthesia workstation breathing system, the exhaled gases move through the hotplate and cross the soda lime once before mixing with the cold and dry FGF. The mixed gases are then pulled by the plunger to fill the ventilator. After opening the inspiratory valve, the ventilator plunger sends the gaseous mixture to the inspiratory limb of the respiratory circuit, where it is warmed a second time by the built-in hotplate. The temperature and humidity of the inhaled gases coming from this anesthesia machine have not yet been investigated. It may be expected that the hotplate would warm the inhaled gases coming from this workstation.

The Primus anesthesia workstation (Dräger, Lübeck, Germany) also has a built-in hotplate; however, unlike the Fabius GS Premium, only the expiratory limb is warmed^14^. A study using this machine with a low-flow breathing system showed a lower temperature (25.3 ± 1.4°C) and AH (20.5 ± 3.6 mg H_2_O.L^-1^) of the inspired gas after 120 minutes of connection of the patient to the breathing circuit^14^.

The minimum AH values of the inhaled gases should be 30 mg H_2_O.L^-1^ for patients under tracheal intubation in intensive care unit^19,20^ and 20 mg H_2_O.L^-1^ in patients undergoing general anesthesia with tracheal intubation to avoid dehydration of the respiratory tract^19^.

**2-STUDY HYPOTHESIS**

Humidification and heating of the gases in the breathing circuit from low-flow Fabius GS Premium Dräger anesthesia workstation may not be enough to provide the minimum humidity level of the inspired gases, which can be directly influenced by the OR temperature. We will test the hypothesis that the use of a HME, which retains heat and moisture from exhaled gas by the patient, and the use of TI of the branches of the breathing circuit may increase the humidity and temperature values of the inhaled gas.

**3-OBJECTIVES**

The objectives of this study are to evaluate the temperature and humidity of the inhaled gases from a low-flow breathing system of the Dräger Fabius GS Premium® anesthesia workstation and to compare the effects of adding TI or HME to the breathing circuit on the temperature and humidity of the inhaled gases from this anesthesia workstation.

**4-PATIENTS AND METHODS**

This study will be performed at Sao Paulo State University Hospital (UNESP, Botucatu, Brazil) and after approval by the Institutional Review Board (Human Research Ethics Committee of the Botucatu Medical School-UNESP), written informed consent will be obtained from all of the patients included in the study. We will evaluate 44 adult (18 to 64 years old) afebrile (T < 37°C) women with a body mass index from 20 to 30 kg.m^-^² with ASA physical status I or II and scheduled for elective open abdominal gynecologic surgery (hysterectomy or oophorosalpingectomy) with at least 2 hours of anesthesia duration. Patients with signs and symptoms of pulmonary or cardiac disease or a body mass index > 30 kg.m^-^² will be excluded from the study.

**4.1 Study design**

The patients will be randomly allocated by sequentially numbered sealed envelope assignment into four groups (11 women per group), according to the use of a low-flow breathing circuit with TI or an HME, as follows: control group, without TI or HME; TI group, with TI and without HME; HME group, without TI and with HME; and TIHME group, with TI and HME. An anesthesiologist who will be not involved in the perioperative management of the patients will print 44 group identification tags (11 for each group) and will place these tags into 44 opaque envelopes (one identification tag per envelope). The same anesthesiologist then will seal, mix and sequentially number the envelopes in ascending order. Just before each anesthesia induction, one envelope will be opened following the sequential order to identify the study group.

**4.2 Anesthesia protocol**

A Dräger Fabius GS Premium anesthesia workstation will be used in all cases. Before each anesthetic procedure, the tubes of the anesthesia breathing system will be replaced by clean and dry silicone corrugated tubes of the same length (1.5 m; Dräger Medical, Lübeck, Germany). The CO_2_ canister (1.5 L) of the anesthesia machine will be filled with fresh soda lime (Drägersorb 800 Plus, Dräger, Lübeck, Germany) before each case. The tubing system, including the CO_2_ absorber, has an internal volume of 4.5 L. In the TI and TIHME groups, the branches of the breathing circuit will be covered by three layers of aluminum sheets. In the HME and TIHME groups, a hygroscopic HME (Venticaire model 038-41-355; Flexicare Medical Limited, Mountain Ash, UK) will be placed between the Y-piece of the breathing circuit and the tracheal tube.

Upon arrival to the OR, and after 8 hours of fasting, the patients will receive standard clinical monitoring with an electrocardiogram (D_II_ and V_5_ leads), peripheral oxygen saturation (SpO_2_), noninvasive arterial blood pressure measurements, a neuromuscular blockade monitor (TOF-watch SX, GPV Elbau Electronics A/S, Aars, Denmark) and a cerebral state index (CSI®) (Danmeter, Biometer International, Odense, Denmark).

An intravenous (IV) line will be inserted with a 20 or 18-G catheter and the patients will receive 3 mg midazolam via IV. Fluid deficits will be replaced with lactated Ringer’s solution at 10 mL.kg^-1^.h^-1^. In all of the patients, the fluids will be maintained at the OR temperature. All of the patients will receive active skin-surface warming with a specific blanket on the lower limbs using a warming device (Bair Hugger®, model 750, Arizant Healthcare, Minneapolis, MN) set to delivery forced-air at 43°C following anesthesia induction and lasting until the end of the surgery.

Total intravenous anesthesia will be induced with a target controlled infusion (TCI) of remifentanil (50 µg.mL^-1^), using a computer-controlled infusion pump (Alaris, Cardinal Health, Rolle, Switzerland) programmed with the Minto adult pharmacokinetic model for remifentanil^21^.Two minutes later, a TCI of 1% propofol will be administered using a computer-controlled infusion pump (Diprifusor, Fresenius Vial, Brezens, France) programmed with the Marsh adult pharmacokinetic model for propofol^22^. The initial target remifentanil plasma concentration will be set at 6.0 ng.mL^-1^, and the propofol plasma concentration will be set at 4.0 µg.mL^-1^. An IV bolus of cisatracurium besylate (0.15 mg.kg^-1^) will be given to facilitate orotracheal intubation. The target propofol and remifentanil concentrations will be maintained between 3.0-5.0 µg.mL^-1^ and 4.0-8.0 ng.mL^-1^, respectively, to maintain a systemic arterial blood pressure and cardiac rate within ± 20% of baseline and CSI values between 40 and 50. Additional cisatracurium doses (0.05 mg.kg^-1^) will be provide, when necessary.

After tracheal intubation, a FGF of 2.0 L.min^-1^ (1.0 L.min^-1^ of O_2_ in 1.0 L.min^-1^ of air) will be supply to the circle breathing during the first 5 minutes and then adjusted to 1.0 L.min^-1^ (0.5 L.min^-1^ of O_2_ in 0.5 L.min^-1^ of air). The lungs will be mechanically ventilated using the volume-controlled mode of the Fabius anesthesia workstation with a tidal volume of 8 mL.kg^-1^. The respiratory rate will be adjusted to maintain an end-tidal carbon dioxide pressure (PetCO_2_) of approximately 35 mm Hg. The inspiratory and expiratory oxygen concentrations, PetCO_2_ and ventilation variables will be monitored with the Fabius built-in monitor.

**4.3 Measurement of temperature and humidity of the gases**

The primary outcomes temperature and humidity of the gases will be intermittently measured using a rapidly responding electronic digital thermo-hygrometer (Vaisala Humicap® Hand-Held Humidity and Temperature Meter HM 70, Helsinki, Finland) that will be connected by a T-piece between the Y-piece of the breathing circuit and the tracheal tube in the control and TI groups, and between the HME and the tracheal tube in the HME and TIHME groups, and it will be connected to the inspiratory limb outlet close to the anesthesia workstation in all of the groups. The thermo-hygrometer operates on a capacitive principle and has a reported accuracy of ± 2.0% for RH and ± 0.2°C for temperature. The temperature and humidity of the gases are altered with the respiratory cycle phases and are lower in the inspiratory phase. We will record the minimal temperature and RH values averaged over 4 respiratory cycles after 10, 30, 60, 90, and 120 minutes of connection between the patient and the respiratory circuit after tracheal intubation. The AH values will be calculated using the thermo-hygrometer software with the formula AH = (3.939 + 0.5019T + 0.00004615T^2^ + 0.0004188T^3^) × RH/100, in which AH is the absolute humidity (mg H_2_O.L^-1^), T is the temperature (°C), and RH is the relative humidity (%).

The secondary outcome intraoperative distal esophageal (core) temperature will be measured after tracheal intubation using a thermocouple sensor (Mon-a-therm 90,044®, Mallinckrodt Medical, Veracruz, Mexico). The thermocouple sensors for the esophagus and OR temperatures will be attached to a 2-channel electronic thermometer (4070, Mallinckrodt Medical, St. Louis, MO).

Postoperative analgesia will comprise 0.1 mg.kg^-1^ morphine, 100 mg tramadol and 100 mg cetoprofen IV, which will be provided 15 minutes before the end of the surgery. The residual neuromuscular blockade will be reversed with 30 µg.kg^-1^ of neostigmine and 10 µg.kg^-1^ of atropine IV, if necessary. Tracheal extubation will be performed after full reversal of the neuromuscular blockade. All of the patients will be transferred to the Postanesthesia Care Unit.

**4.4 Statistical analysis**

The sample size of the groups was calculated based on data found in the literature regarding the humidification of gases during anesthesia^7,17^ and assuming an AH minimum detectable difference of 5.0 mg H_2_O.L^-1^ among the groups, with an expected standard deviation (SD) of residuals of ± 3.0 mg H_2_O.L^-1^ as significant. For 80% power and considering the risk of a type I error as α of 0.05 or less and of committing type II error as β of 0.20 or less, 9 patients in each group would be required. Since missing data of patients can occur during the study, 11 patients in each group will be enrolled.

The normal distribution of the data will be confirmed using Lilliefors tests. The anthropometric variables will be compared among groups by analysis of variance (ANOVA). The AH, RH, and temperature values will be compared among the groups at different time points by repeated measured ANOVA (Profile Analysis). In this analysis, the following hypotheses will be tested: there was no interaction between groups and time points, and there was no difference between mean groups over time. This analysis will be followed by Tukey’s test for pairwise comparisons. Pearson’s coefficient will be used for the correlation analysis between the OR and inhaled gas temperatures in all of the groups.

The statistical analyses will be performed using the Statistical Package for the Social Sciences (Windows Software, version 17.1; SPSS Inc., Chicago, IL). For all analyses, *P* < 0.05 will be considered statistically significant.

**5-REFERENCES**

1. Van Oostdam JC, Walker DC, Knudson K, Dirks P, Dahlby RW, Hogg JC. Effect of breathing dry air on structure and function of airways. J Appl Physiol 1986; 61: 312-7.

2. Chalon J, Ali M, Ramanathan S, Turndorf H. The humidification of anaesthetic gases. its importance and control. Can Anaesth Soc J 1979; 26: 361-6.

3. Williams R, Rankin N, Smith T, Galler D, Seakins P. Relationship between the humidity and temperature of inspired gas and the function of the airway mucosa. Crit Care Med 1996; 24: 1920-9.

4. Carson KD. Humidification during anesthesia. Respir Care Clin N Am 1998; 4: 281-99.

5. Shelly MP, Lloyd GM, Park GR. A review of the mechanisms and methods of humidification of inspired gases. Intensive Care Med 1988; 14: 1-9.

6. Shelly MP. Inspired gas conditioning. Respir Care 1992; 37: 1070-80.

7. Barra Bisinotto FM, Braz JR, Martins RH, Gregorio EA, Abud TM. Tracheobronchial consequences of the use of heat and moisture exchangers in dogs. Can J Anaesth 1999; 46: 897-903.

8. Brock-Utne JG. Humidification in paediatric anaesthesia. Paediatr Anaesth 2000; 10: 117-9.

9. Martins RHG, Braz JRC, Defaveri J, Cury PR. Estudo da umidificação e do aquecimento dos gases durante a ventilação mecânica no cão. Rev Bras Otorrinolaringol 1996; 62: 206-218.

10. Johansson A, Lundberg D, Luttropp HH. The effect of heat and moisture exchanger on humidity and body temperature in a low-flow anaesthesia system. Acta Anaesthesiol Scand 2003; 47: 564-8.

11. Hendrickx JF, De Wolf A. Special aspects of pharmacokinetics of inhalation anesthesia. Handb Exp Pharmacol 2008: 159-86.

12. Baker AB. Low flow and closed circuits. Anaesth Intensive Care 1994; 22: 341-2.

13. Dorsch JA DS. The Circle System, Understanding Anesthesia Equipment, 4th Edition. Edited by Wilkins W. Baltimore, 1999, p 229-269.

14.de Castro J, Jr., Bolfi F, de Carvalho LR, Braz JR. The temperature and humidity in a low-flow anesthesia workstation with and without a heat and moisture exchanger. Anesth Analg 2011;113:534-8.

15. Torres MLA, Carvalho CJA, Bello CN, Cremonesi E, Mathias RS. Sistemas respiratórios valvulares com absorção de CO2: capacidade de aquecimento e umidificação dos gases inalados em três tipos de montagens utilizadas em aparelhos de anestesia no Brasil. Rev Bras Anestesiol 1997; 47: 89-100.

16. Hunter T, Lerman J, Bissonnette B. The temperature and humidity of inspired gases in infants using a pediatric circle system: effects of high and low-flow anesthesia. Paediatr Anaesth 2005; 15: 750-4.

17. Gorayb SB, Braz JR, Martins RH, Modolo NS, Nakamura G. Inhaled gases humidification and heating during artificial ventilation with low flow and minimal fresh gases flow. Rev Bras Anestesiol 2004; 54: 20-36.

18. Lemmens HJ, Brock-Utne JG. Heat and moisture exchange devices: are they doing what they are supposed to do? Anesth Analg 2004; 98: 382-5.

19. Wilkes AR. Heat and moisture exchangers and breathing system filters: their use in anaesthesia and intensive care. Part 1 - history, principles and effciency. Anaesthesia 2011; 66: 31-9.

20. International Organization for Satandardization. Respiratory tract humidifiers for medical use—Particular requirements for respiratory humidification systems. Geneva, Switzerland, International Organization for Standardization, ISO 8115 2007.

21.Minto CF, Schnider TW, Shafer SL. Pharmacokinetics and pharmacodynamics of remifentanyl. II. Model application. Anesthesiology 1997; 86: 24-33.

22.Marsh B, White M, Morton N, Kenny GN. Pharmacokinetic model driven infusion of propofol in children. Br J Anaesth 199;1 67: 41-8.
