# Supplementary material for: The Humidity in a Low-Flow Dräger Fabius Anesthesia Workstation with or without Thermal Insulation or a Heat and Moisture Exchanger: A Prospective Randomized Clinical Trial
Source: PLoS One. 2017 Jan 27;12(1):e0170723. doi: 10.1371/journal.pone.0170723 (PMC5271343; doi:10.1371/journal.pone.0170723)
Supplement: S2 Protocol — (DOC) [file pone.0170723.s003.doc]

Protocolo de Pesquisa Enviado ao Comitê de Ética em Pesquisa do Hospital das Clínicas da Faculdade de Medicina de Botucatu, UNESP

Umidificação e aquecimento do gás inalado em estação de anestesia com baixo fluxo de gases utilizando ou não isolamento térmico do circuito ventilatório **e permutador de calor e umidade**

**Sergius Arias Rodrigues de Oliveira**

Aluno do Curso de Mestrado do Programa de Pós-Graduação em Anestesiologia da Faculdade de Medicina de Botucatu, UNESP

**Orientador**

**José Reinaldo Cerqueira Braz**

Professor Titular do Departamento de Anestesiologia da Faculdade de Medicina de Botucatu, Universidade Estadual Paulista (UNESP – Botucatu)

**Outubro de 2011**

1. **INTRODUÇÃO**

Em circunstâncias normais, as vias aéreas superiores aquecem e umidificam o ar inspirado. Durante a inspiração, o ar chega aos alvéolos à temperatura central, que é de 37ºC, e com umidade absoluta de 44 mg H2O.L-1  e relativa de 100%1. O fluxo de gases utilizado durante a anestesia é frio e seco. Quando se emprega o tubo traqueal durante a anestesia, as funções do nariz e das demais vias aéreas superiores são anuladas. Caso a umidificação e o aquecimento dos gases inalados não sejam adequados, ocorrerá prejuízo funcional da mucosa das vias aéreas, com alteração do movimento ciliar e aumento da viscosidade do muco, provocando endurecimento e incrustações das secreções, aumento da resistência das vias aéreas, alterações da relação ventilação-perfusão, microatelectasias, diminuição da complacência pulmonar e da capacidade residual funcional, alterações da função do surfactante pulmonar, reações inflamatórias, ulcerações nas mucosas e desidratação, predispondo à formação de rolhas que obstruem as vias aéreas, principalmente as de pequeno calibre, e os tubos traqueais2-6. Assim, durante a ventilação artificial realizada sob intubação traqueal, o aquecimento e a umidificação dos gases inspirados são essenciais para assegurar a integridade das vias aéreas e a preservação da função mucociliar e das trocas gasosas4,7.

Há discordância em relação aos valores ideais de aquecimento e umidificação dos gases inalados pelos pacientes durante a anestesia geral, mas é necessário o estabelecimento de limites de aquecimento e umidificação do gás inspirado para que não ocorram lesões da mucosa respiratória8. Verificou-se, em estudo experimental em cães submetidos a intubação traqueal e ventilação mecânica durante três horas, os efeitos do aquecimento e umidificação dos gases inspirados, de acordo com as alterações histológicas do epitélio da mucosa da árvore traqueobrônquica à microscopia óptica. Os autores concluíram que a temperatura e a umidade absoluta dos gases inspirados não devem ultrapassar, respectivamente, 36ºC e 36 mg H2O.L-1 de gás inspirado, e não devem ter temperatura inferior a 27ºC ou apresentarem umidade absoluta inferior a 23 mg H2O.L-1 9.

A utilização de baixo fluxo de gases frescos durante a anestesia geral inalatória aumentou muito nos últimos anos, por apresentar importantes vantagens, como menor consumo de anestésico inalatório, umidificação e aquecimento mais efetivo do gás inspirado e diminuição importante da poluição ambiental10-12. No entanto, a utilização de baixo fluxo de gases frescos apresenta desvantagens, como a necessidade de maior atenção e cuidados do anestesiologista, pela impossibilidade de rápida alteração da concentração inspirada do anestésico inalatório, maior possibilidade de produção de hipercarbia pela exaustão mais rápida do sistema de absorção de dióxido de carbono e o eventual acúmulo de gases indesejados, como monóxido de carbono, acetona, metano, além de metabólitos tóxicos dos agentes anestésicos, argônio e nitrogênio12, exigindo a realização de “lavagem” periódica do sistema com alto fluxo de gases frescos por alguns minutos.

A redução do fluxo de gás fresco leva ao maior aproveitamento de calor e umidade do ar expirado e dos que são gerados no reservatório do absorvedor canister, por meio da reação de neutralização do CO2 da mistura exalada pela cal sodada, que é exotérmica e leva à formação de água 13-15. Vários autores utilizaram baixo fluxo de gases frescos com bons resultados, porém com eficiência variável em relação ao aproveitamento do calor e umidade10,14-17, sobretudo quando associados aos permutadores de calor e umidade10,14. As diferenças encontradas podem ser explicadas por alterações nas montagens dos sistemas respiratórios empregados, pelos diferentes métodos utilizados para medição de umidade presente nos gases inspirados, por permutadores com materiais e performances diferentes18,19 e os diferentes fluxos utilizados na técnica de baixo fluxo de gases frescos10.

A temperatura da sala cirúrgica influencia diretamente a temperatura e, consequentemente, a umidade do gás inspirado no sistema ventilatório de diferentes aparelhos de anestesia. Isto ocorre pelo baixo isolamento térmico dos tubos de silicone empregados nos circuitos respiratórios14,15.

A Estação de Anestesia Fabius GS Premium da Dräger (Lübeck, Alemanha) possui sistema ventilatório que recebe aquecimento adicional por meio de placa aquecida. Assim, o gás expirado pelo paciente passa inicialmente pela válvula expiratória, que nessa fase encontra-se aberta. Em seguida, pela placa aquecida, passando a seguir e uma única vez pela cal sodada em direção ao reservatório de gás fresco (balão). Em seguida, o gás expirado, juntamente com o fluxo adicional de gases frescos, é “puxado” pela movimentação do pistão do respirador para o preenchimento desse último. A seguir o fluxo misto de gases passa para a parte inspiratória, que também recebe aquecimento por placa aquecida. Essas características do aparelho podem aumentar os valores de temperatura e umidade dos gases inalados. Ainda não há informação, na literatura ao nosso alcance, sobre os níveis de umidificação e aquecimento fornecidos por esse sistema ao gás inalado.

A estação de anestesia Primus da Dräger (Alemanha) também possui sistema ventilatório semelhante ao do Fabius GS Premium com placa aquecida no sistema, mas somente no ramo expiratório14. Um estudo utilizando essa estação de anestesia e baixo fluxo de gases frescos (1 L.min-1) obtiveram valores de temperatura de 25,3±1,4°C e de umidade absoluta de 20,5 ± 3,6 mg H2O.L-1 do gás inalado após 120 min de anestesia14.

Os valores mínimos de umidade absoluta nos sistemas de umidificação devem ser de 30 mg H2O.L-1 para pacientes sob intubação traqueal em unidade de terapia intensiva19,20 e de 20 mg H2O.L-1 em pacientes sob anestesia geral19.

1. **HIPÓTESE DE ESTUDO**

A capacidade de umidificação e aquecimento da estação de anestesia Fabius GS Premium da Dräger (Alemanha), utilizando técnica de baixo fluxo de gases pode não ser suficiente para colocar o gás do ramo inspirado do circuito ventilatório em condições de ser inalado dentro do valor mínimo exigido, além de poder ser influenciada diretamente pela temperatura da sala de operação. Testaremos as hipóteses de que a utilização de um permutador de umidade e temperatura que retém o calor e a umidade do gás exalado pelo paciente, ou o uso de isolamento térmico do circuito ventilatório, poderão aumentar os valores de umidade e temperatura do gás inalado.

1. **OBJETIVOS**

O estudo terá como objetivos avaliar a eficiência do sistema ventilatório circular da estação de anestesia Dräger Fabius GS Premium, quanto à capacidade de aquecimento e umidificação dos gases inalados durante a anestesia, utilizando-se baixo fluxo de gases frescos (1L.min-1), e comparar os efeitos do isolamento térmico do sistema ventilatório da estação de anestesia Fabius e a introdução de permutador de calor e umidade no aquecimento e umidificação dos gases inalados.

1. **PACIENTES E MÉTODO**

Após a aprovação do protocolo do estudo pelo Comitê de Ética do Hospital das Clínicas da Faculdade de Medicina de Botucatu da UNESP e obtido consentimento por escrito, serão estudados 44 pacientes adultos, sexo feminino, de 18 a 64 anos, afebril (T < 37°C), com índice de massa corporal de 20 a 30 kg/m2 com classificação de estado físico ASA I-II, submetidos a cirurgia abdominal ginecológica (histerectomia ou ooforossalpingectomia) , com duração de, pelo menos, 120 minutos.

Os critérios de exclusão das pacientes serão sinais e sintomas de doença cardíaca ou pulmonar e índice de massa corpórea superior a 30 kg/m2.

**4.1 Grupos Experimentais**

As pacientes serão distribuídas, de forma aleatória, pela abertura de envelopes lacrados em quatro grupos de 11 pacientes, que diferenciar-se-ão de acordo com o uso ou não de permutador de calor e umidade e de isolamento térmico do circuito ventilatório, durante ventilação com baixo fluxo de gases frescos como segue:

**GRUPO Controle**:sem a utilização de permutador de calor e umidade ou isolamento térmico*;* **GRUPO BFP**:utilização de permutador de calor e umidade, mas sem isolamento térmico*;* **GRUPO BFI**: isolamento térmico do circuito ventilatório mas sem permutados de calor e umidade;e **GRUPO BFPI**: com permutador de calor e umidade e isolamento térmico do circuito ventilatório*.* Um anestesiologista não envolvido no estudo e no manuseio das pacientes preparará 44 papéis de identificação (11 para cada grupo) que, em seguida, serão colocados em 44 envelopes foscos, com uma identificação por envelope. O mesmo anestesiologista, após o fechamento e mistura dos envelopes, numerará sequencialmente os envelopes em ordem ascendente Antes de cada indução da anestesia, um envelope será aberto, seguindo a ordem sequencial, para identificação do grupo de estudo.

A estação de anestesia Dräger Fabius GS Premium será utilizada em todos os procedimentos anestesiológicos. Antes de cada anestesia,os tubos corrugados de silicone secos e limpos, com 1,50 m de comprimento cada, serão trocados. O restante do espaço morto artificial é constituído pelo reservatório (canister) com o absorvedor de CO2 (cal sodada) utilizado (Drägersorb® 800 plus, Dräger, Alemanha), que está situado no ramo expiratório e tem capacidade de 1,5 L. O absorvedor de CO2 também será trocado antes da realização de cada anestesia. Os sistemas de tubos corrugados e o canister do absorvedor têm volume interno total de 4,5 L. Nos grupos BFI e BFPI, os tubos corrugados do sistema ventilatório serão recobertos com três folhas de papel alumínio e nos grupos BFP e BFPI será colocado o permutador de calor e umidade Venticaire (model 038-41-355; Flexicare Medical Limited, Mountain Ash, UK) entre o tubo em “Y”do circuito ventilatório e o tubo traqueal.

. **4.2 Procedimento Anestesiológico**

Após permanecerem em jejum alimentar por 8 horas, as pacientes, ao chegarem à SO, terão punção venosa em um dos braços com cateter 18G ou 20G para sedação com midazolam, na dose de 3 mg, e realização de terapia fluida com 10 ml.kg-1.h-1 de Ringer com lactato (RL) em temperatura ambiente.

A monitorização inicial incluirá eletrocardioscospia (derivações DII e V5), saturação periférica da hemoglobina pelo oxigênio (SpO2) e pressão arterial não invasiva (PANI), monitorização do nível de hipnose pelo índice de estado cerebral (CSI®) no aparelho de eletroencefalograma micro processado da Danmeter (Biometer International, Odense, Dinamarca) e monitorização do bloqueio neuromuscular (TOF-watch SX, GPV Elbau Electronics A/S, Aars, Dinamarca). As pacientes receberão aquecimento por ar forçado nos membros inferiores desde o início da anestesia até o final do procedimento cirúrgico. Utilizar-se-á manta térmica específica para membros inferiores, com temperatura ajustada para 43°C proveniente de aparelho específico (Bair Hugger®, modelo 750, Arizant Healthcare, Minneapolis, EUA).

Para a indução e manutenção da anestesia, utilizar-se-á a técnica venosa total alvo-controlada. Iniciar-se-á com o opioide remifentanil (50 µg/ml), infundido por bomba de infusão Alaris® PK (Cardinal Health, Rolle, Suíça), programada com o modelo farmacocinético para adultos de Minto21, na concentração plasmática predita inicial de 6 ng.ml-1. Dois minutos após, será administrado propofol (1%), infundido com o aparelho Diprifusor® (Fresenius Vial, Brezens França) programado com o modelo farmacocinético de Marsh22, com concentração plasmática inicial predita de 4 ug.ml-1, para produção de inconsciência. Será administrado, em seguida, o besilato de cisatracúrio (150 µg.kg-1, IV) para facilitar a intubação orotraqueal. A manutenção da anestesia será feita com ajustes nas concentrações-alvo de propofol (3.0-5.0 µg.mL-1) e remifentanil (4.0-8.0 ng.mL-1), objetivando índices de estado cerebral de 40 a 60, e alterações hemodinâmicas de pressão arterial e frequência cardíaca de ± 20% dos valores imediatamente anteriores à indução anestésica. Doses adicionais de cisatracúrio (0.05 mg.kg-1) serão administradas quando necessário.

Após a intubação traqueal, o FGF será mantido em 2 L.min-1 por período de 5 minutos, para completo preenchimento dos gases dentro do circuito ventilatório, para em seguida, ser diminuído para 1 L.min-1 (ar comprimido - 0,5 L.min-1 + O2 - 0,5 L.min-1). Em seguida, a paciente será conectada ao sistema circular de ventilação da estação de anestesia e iniciada ventilação mecânica controlada a volume (8 ml.kg-1). A frequência respiratória fserá ajustada para manutenção de capnometria próxima de 35 mm Hg. As análises das concentrações de O2 inspirado e expirado e da pressão inspiratória e expiratória final de CO2 (PETCO2), e os parâmetros ventilatórios serão feitas pelo biomonitor integrado à estação de anestesia.

**4.3 Medidas de temperatura e umidade dos gases**

Os atributos primários de temperatura e umidade dos gases serão intermitentemente mensurados, utilizando-se termo-higrômetro eletrônico de rápida resposta digital (Vaisala Humicap® Hand-Held Humidity and Temperature Meter HM 70, Helsinki, Finlândia), conectando-se o seu sensor, por meio de peça em T, inicialmente na saída do circuito ventilatório, junto à estação de trabalho e, em seguida, entre o tubo “Y” do circuito respiratório e o tubo traqueal nos grupos controle e BFI e entre o permutador de calor e umidade e o tubo traqueal nos grupos BFP e BFPI. O termo-higrômetro capacitivo tem acurácia de ± 2.0% para a umidade relativa e de ± 0.2°C para a temperatura. A temperatura e umidade dos gases se alteram de acordo com a fase do ciclo respiratório e são menores na fase inspiratória. Serão registrados os valores médios mínimos durante o período de quatro ciclos respiratórios após 10, 30, 60, 90, e 120 minutos de conexão do tubo traqueal da paciente com o circuito respiratório. Os valores de UA mostrados na tela são calculados pelo *software* do termo-higrômetro segundo a seguinte fórmula: UA = (3,939 + 0,5019T + 0,00004615T2 + 0,0004188T3) x UR / 100, onde T é a temperatura (°C) e UR a umidade relativa (%).

Os valores do atributo secundário temperatura esofagiana serão medidos após a intubação traqueal utilizando um sensor esofágico (Mon-a-therm 90,044®, Mallinckrodt Medical, Veracruz, México). Os sensores de temperatura esofágica e ambiente serão conectados a um termômetro de 2 canais da Mallinckrodt Medical (St. Louis, EUA).

A analgesia pós-operatória será feita 15 minutos antes do término da cirurgia com morfina 0.1 mg.kg-1, tramadol 100 mg e cetoprofeno 100mg IV. Bloqueio neuromuscular residual será revertido com sulfato de atropina-0,010 mg.kg­1 e neostigmina - 0,03 mg.kg­1. Após extubação traqueal, as pacientes serão, a seguir, encaminhadas à Sala de Recuperação Pós-Anestésica, onde permanecerão até a alta para o leito de enfermaria.

**4.4 Análise estatística**

O tamanho amostral foi determinado pela literatura relacionada à umidificação dos gases inalados durante a anestesia7,17 e considerando-se diferença esperada entre as médias dos grupos da umidade absoluta de 5 mg H2O/L e desvio padrão de ± 3,0 mg H2O/L. Considerou-se um poder do teste (β) de 80% (erro tipo II) e erro tipo I (alfa) de 5%. Determinou-se a necessidade de um número mínimo de 9 pacientes por grupo de estudo. Como poderá ocorrer perda de dados de pacientes, serão estudadas 11 pacientes por grupo de estudo.

Após a coleta de dados ser concluída e confirmação de distribuição normal dos valores obtidos, atestada pelo teste de Lilliefors, utilizaremos a análise de variância (ANOVA) para comparação dos dados antropométricos entre os grupos. Para as variáveis quantitativas será utilizado teste do qui-quadrado. Para as variáveis cujo interesse será comparar grupos e momentos, será utilizada a análise de perfil. Nesta análise, as hipóteses testadas serão as seguintes: não há interação entre grupos e tempos e não há diferença significante entre as médias dos grupos ao longo do tempo. Nos casos de significância, serão feitas comparações pelo teste de Tukey. O coeficiente de Pearson será utilizado para verificação de correlação entre as temperaturas das sala de operação e do gás inalado nos grupos estudados. As análises estatisticas serão realizadas no *Statistical Package for the Social Sciences (Windows Software, version 17.1; SPSS Inc., Chicago, IL)* em todas as análises, *P* < 0.05 será considerado estatisticamente significante.

**5. REFERÊNCIAS**

1. Van Oostdam JC, Walker DC, Knudson K, Dirks P, Dahlby RW, Hogg JC. Effect of breathing dry air on structure and function of airways. J Appl Physiol 1986; 61: 312-7.

2. Chalon J, Ali M, Ramanathan S, Turndorf H. The humidification of anaesthetic gases. its importance and control. Can Anaesth Soc J 1979; 26: 361-6.

3. Williams R, Rankin N, Smith T, Galler D, Seakins P. Relationship between the humidity and temperature of inspired gas and the function of the airway mucosa. Crit Care Med 1996; 24: 1920-9.

4. Carson KD. Humidification during anesthesia. Respir Care Clin N Am 1998; 4: 281-99.

5. Shelly MP, Lloyd GM, Park GR. A review of the mechanisms and methods of humidification of inspired gases. Intensive Care Med 1988; 14: 1-9.

6. Shelly MP. Inspired gas conditioning. Respir Care 1992; 37: 1070-80.

7. Barra Bisinotto FM, Braz JR, Martins RH, Gregorio EA, Abud TM. Tracheobronchial consequences of the use of heat and moisture exchangers in dogs. Can J Anaesth 1999; 46: 897-903.

8. Brock-Utne JG. Humidification in paediatric anaesthesia. Paediatr Anaesth 2000; 10: 117-9.

9. Martins RHG, Braz JRC, Defaveri J, Cury PR. Estudo da umidificação e do aquecimento dos gases durante a ventilação mecânica no cão. Rev Bras Otorrinolaringol 1996; 62: 206-218.

10. Johansson A, Lundberg D, Luttropp HH. The effect of heat and moisture exchanger on humidity and body temperature in a low-flow anaesthesia system. Acta Anaesthesiol Scand 2003; 47: 564-8.

11. Hendrickx JF, De Wolf A. Special aspects of pharmacokinetics of inhalation anesthesia. Handb Exp Pharmacol 2008: 159-86.

12. Baker AB. Low flow and closed circuits. Anaesth Intensive Care 1994; 22: 341-2.

13. Dorsch JA DS. The Circle System, Understanding Anesthesia Equipment, 4th Edition. Edited by Wilkins W. Baltimore, 1999, p 229-269.

14.de Castro J Jr., Bolfi F, de Carvalho LR, Braz JR. The temperature and humidity in a low-flow anesthesia workstation with and without a heat and moisture exchanger. Anesth Analg 2011;113:534-8.

15. Torres MLA, Carvalho CJA, Bello CN, Cremonesi E, Mathias RS. Sistemas respiratórios valvulares com absorção de CO2: capacidade de aquecimento e umidificação dos gases inalados em três tipos de montagens utilizadas em aparelhos de anestesia no Brasil. Rev Bras Anestesiol 1997; 47: 89-100.

16. Hunter T, Lerman J, Bissonnette B. The temperature and humidity of inspired gases in infants using a pediatric circle system: effects of high and low-flow anesthesia. Paediatr Anaesth 2005; 15: 750-4.

17. Gorayb SB, Braz JR, Martins RH, Modolo NS, Nakamura G. Inhaled gases humidification and heating during artificial ventilation with low flow and minimal fresh gases flow. Rev Bras Anestesiol 2004; 54: 20-36.

18. Lemmens HJ, Brock-Utne JG. Heat and moisture exchange devices: are they doing what they are supposed to do? Anesth Analg 2004; 98: 382-5.

19. Wilkes AR. Heat and moisture exchangers and breathing system filters: their use in anaesthesia and intensive care. Part 1 - history, principles and effciency. Anaesthesia 2011; 66: 31-9.

20. International Organization for Satandardization. Respiratory tract humidifiers for medical use—Particular requirements for respiratory humidification systems. Geneva, Switzerland, International Organization for Standardization, ISO 8115 2007.

21.Minto CF, Schnider TW, Shafer SL. Pharmacokinetics and pharmacodynamics of remifentanyl. II. Model application. Anesthesiology 1997; 86: 24-33.

22.Marsh B, White M, Morton N, Kenny GN. Pharmacokinetic model driven infusion of propofol in children. Br J Anaesth 199;1 67: 41-8.
